# Biomimetic Approaches for Separated Regeneration of Sensory and Motor Fibers in Amputee People: Necessary Conditions for Functional Integration of Sensory–Motor Prostheses With the Peripheral Nerves

**DOI:** 10.3389/fbioe.2020.584823

**Published:** 2020-11-03

**Authors:** Atocha Guedan-Duran, Nahla Jemni-Damer, Irune Orueta-Zenarruzabeitia, Gustavo Víctor Guinea, José Perez-Rigueiro, Daniel Gonzalez-Nieto, Fivos Panetsos

**Affiliations:** ^1^Neuro-computing and Neuro-robotics Research Group, Complutense University of Madrid, Madrid, Spain; ^2^Innovation Group, Institute for Health Research San Carlos Clinical Hospital (IdISSC), Madrid, Spain; ^3^Department of Biomedical Engineering, Tufts University, Medford, MA, United States; ^4^Center for Biomedical Technology, Universidad Politécnica de Madrid, Madrid, Spain; ^5^Department of Material Science, Civil Engineering Superior School, Universidad Politécnica de Madrid, Madrid, Spain; ^6^Biomedical Research Networking Center in Bioengineering, Biomaterials and Nanomedicine (CIBER-BBN), Madrid, Spain; ^7^Silk Biomed SL, Madrid, Spain

**Keywords:** biomolecules, biomaterials, neurotrophins, prostheses, neurotrophic factors, protein, mRNA

## Abstract

The regenerative capacity of the peripheral nervous system after an injury is limited, and a complete function is not recovered, mainly due to the loss of nerve tissue after the injury that causes a separation between the nerve ends and to the disorganized and intermingled growth of sensory and motor nerve fibers that cause erroneous reinnervations. Even though the development of biomaterials is a very promising field, today no significant results have been achieved. In this work, we study not only the characteristics that should have the support that will allow the growth of nerve fibers, but also the molecular profile necessary for a specific guidance. To do this, we carried out an exhaustive study of the molecular profile present during the regeneration of the sensory and motor fibers separately, as well as of the effect obtained by the administration and inhibition of different factors involved in the regeneration. In addition, we offer a complete design of the ideal characteristics of a biomaterial, which allows the growth of the sensory and motor neurons in a differentiated way, indicating (1) size and characteristics of the material; (2) necessity to act at the microlevel, on small groups of neurons; (3) combination of molecules and specific substrates; and (4) temporal profile of those molecules expression throughout the regeneration process. The importance of the design we offer is that it respects the complexity and characteristics of the regeneration process; it indicates the appropriate temporal conditions of molecular expression, in order to obtain a synergistic effect; it takes into account the importance of considering the process at the group of neuron level; and it gives an answer to the main limitations in the current studies.

## Introduction

The peripheral nervous system (PNS) is responsible for connecting the periphery with the central nervous system, transmitting sensory signals, from the sensors to the brain (afferent) and motor and from the brain to the muscles and glands (efferent). An essential feature of the PNS is the regenerative capacity it presents after suffering damage. Depending on the degree of damage, the number of damaged nerve structures, and the proximity to the nucleus, especially important for its role in the activation of the regenerative machinery (regeneration associated genes), regeneration will be more or less complete. The two main problems were (i) the distance or gap that is generated between the two ends of the damaged nerve and (ii) the intermingled growth of sensory and motor fibers that leads to erroneous reinnervation of the target tissues, sensors, and muscle fibers, respectively (Ramón y Cajal, 1959; Nerves and nerve injuries. By Sunderland, 1969, C.M.G., M.D., B.S., D.Sc., F.R.A.C.S.(Hon.), F.R.A.C.P., F.A.A. (Melbourne). 10 × 7 in. Pp. 116 + xvi, with 197 illustrations. 1968. Edinburgh: E. & S. Livingstone Ltd., £ 12 10s, 1969; Sulaiman and Gordon, 2013).

Therefore, the priority objectives of materials engineering in this specific hot research topic are (i) the development of biomaterials that allow the bridging of the two ends of the damaged nerve; (ii) the provision of these biomaterials of a substrate that guides and supports the regenerating nerves; and (iii) the achievement of a differentiated growth of the sensory and motor nerve fibers, each of them toward its original tissues. For this, it is necessary to have a thorough knowledge of the repair processes of the PNS and the molecular basis of the regeneration of each type of nerve fiber, and we have to become able to manage to incorporate these specific processes in the new biomaterials.

Acquiring this knowledge is a very difficult task because the regeneration processes are extremely complex; the literature is very broad, with different methodologies that are not comparable to each other and results that, in most cases, are very difficult to interpret and use for the development of new therapies. The first big problem is that, for reasons of complexity of the experiments, the studies are neither systematic nor complete. Each one focuses only on some of the tens or hundreds of factors that are involved, but the factors are not the same, and their same form of expression (mRNA, proteins, genes…) is not studied. The task is further complicated because the expression of these factors depends drastically on the area of the nerve being studied (neuron soma, injured tissue, proximal stump, distal stump, target tissue…) and changes over time. All this makes it very difficult to compare the results published in the different articles and even more difficult to synthesize them. However, the most serious problem is the excessive simplification of the studies: there is a huge number of articles on the influence of the different molecules on the regeneration of the peripheral nerves, but this influence is studied considering the molecules in isolation when, as will be explained throughout this article, the regeneration process is very complex and is carried out, thanks to the synergistic action of a large number of molecules, each of which has a temporal expression pattern very different from the others.

All this represents a very big obstacle for scientists who are dedicated to tissue engineering, the development of advanced biomaterials, and cell therapy for the repair of peripheral nerve injury (PNI). Almost all of up-to-day approaches are limited to choosing restricted amounts of biomolecules or cells with regenerative properties, incorporating them into scaffolds and testing to see their efficiency, but obviously, with very poor results.

In this article, (i) we offer an understandable synthesis of the regeneration processes after a PNI and the role that neurotrophic molecules play in those processes; (ii) we create a framework and rationale to be able to engineer therapeutic solutions based on biomaterials and molecules scientifically and non-empirically neurotrophic; and (iii) we propose biomimetic approaches for the development of advanced bio-hybrid devices to be used not only in severe PNI but also in nerve–machine interfaces for amputee people.

## Peripheral Nerves, Nerve Injuries, and Amputations

The PNS is responsible for transmitting sensory information from the periphery and from the internal organs to the central nervous system (spinal cord and/or brain) and for transmitting motor neural activity from the brain and/or spinal cord to the muscle fibers and glands (Flores et al., 2000; Catala and Kubis, 2013).

**FIGURE 1:**
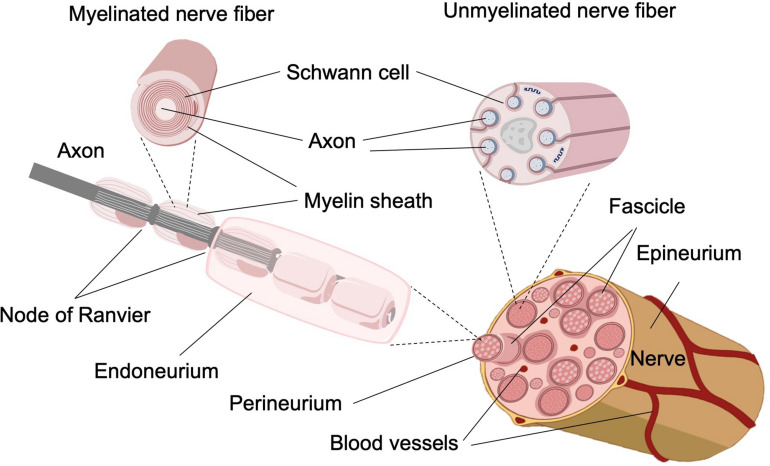
Representative structure of a peripheral nerve. **(Top)** Representation of the functional unit made up of axons wrapped by the Schwann cell. The representation of myelinated and unmyelinated fibers and their differences by the presence of myelin and by the number of axons involved by each Schwann cell can be observed. **(Bottom)** In the peripheral nervous system, each nerve fiber is surrounded by a Schwann cell and the endoneurium individually, consisting of loose connective tissue. In turn, the individual neurons are grouped into bundles, called fascicles, and each of these is surrounded by the perineurium. The epineurium, the outermost layer, is going to be in charge of protecting and unifying the nerve. Finally, we find the presence of the vasa nervorum.

The main architectural elements of the PNS are the axons of the neural cells. Thick ones (Ø > 1 μm), normally associated with motor and exteroceptive or proprioceptive information, are myelinated, which means surrounded by the membranes of several Schwann cells that cover the whole axon, placed one after the other from the soma to the synaptic terminals. The gaps between Schwann cells, the spaces where axons are not wrapped by the myelin sheath, are called Nodes of Ranvier. Thin axons (0.5 < Ø < 1.5 μm), normally associated with temperature and/or pain perception, are not individually myelinated, but rather a single Schwann cell wraps up to 15 or more axons together (Figure 1; Sherman and Brophy, 2005; Griffin and Thompson, 2008; Catala and Kubis, 2013). Each myelinated axon, or group of unmyelinated axons, is surrounded and protected by the endoneurium, a thin cylinder of lax connective tissue made by collagen fibrils, fibroblasts, capillaries, fixed macrophages, and perivascular mast cells; endoneuria are filled with the endoneural fluid, a liquid containing fine proteins equivalent to the cerebrospinal fluid of the central nervous system, within which the axons are suspended (Figure 1).

The axons of each modality are divided in several fascicles, each of them surrounded by the perineurium, a connective tissue denser than the endoneurium, composed of several layers of flattened fibroblasts, enclosed in basal lamina (Stewart, 2003; Brushart, 2011). The basal lamina is a thin layer of extracellular tissue that underlies the epithelium and surrounds muscle fibers, adipocytes, and Schwann cells to separate them from the adjacent connective tissue on both sides and provides strength and elasticity to the peripheral nerve (Bunge et al., 1986; Court et al., 2006). Perineuria tissue is circularly oriented with respect to the direction of the axons (Figure 1; Battista and Lusskin, 1986; Flores et al., 2000).

The total numbers of fascicles are finally covered with the epineurium, a dense tissue sheet longitudinally oriented with respect to the nerve fibers, made up of connective cells, some fat cells, and collagen that holds the fascicles and irrigates the whole structure through the vasa nervorum that run along inside (Figure 1; Adams, 1942; Zochodne, 2018), which constitute the blood–nerve barrier, present at epineurium and endoneurium level where the smallest vessels are present. Formally, the epineurium is the component that defines the peripheral nerve as an organ, as a functional structure built by multifascicular anatomical structures of a high number of unidirectional channels (Figure 1). Depending on the type of information transmitted by the neurons, their soma will be found at the level of the dorsal root ganglia in the case of the sensory neurons (Krames, 2015) and on the ventral horn of the spinal cord in the case of the motor neurons of the PNS (Nógrádi et al., 2011).

The damage of any of the physical components of a peripheral nerve, caused by accidents, military activities, endogenous or exogenous toxins, metabolic diseases, etc., is included on the PNI (Dubový, 2004; Gu et al., 2011). A very special case of PNI are amputations and severe mutilations in which the distal part of a member gets lost together with a part of the nerve as well as with the target organs, sensory organs, and muscles. Amputations are due to accidents or therapeutic surgeries, mostly vascular diabetes in developed countries and war-like actions, infections, or trauma in developing and underdeveloped countries (Dillingham et al., 2002; Olasinde et al., 2002; Abou-Zamzam et al., 2003). Amputations are normally accompanied by neuropathies, by phantom limb symptoms and in the 60 to 80% of the cases also by phantom limb pain (Cuartero et al., 2012; Nikolajsen and Christensen, 2015).

In 2005, it is estimated that there were 1.6 million people suffering from limb amputations in the United States and a similar number in the EU (Mohanna et al., 2003; Ziegler-Graham et al., 2008; Marshall and Stansby, 2010). By 2050, in the United States, the number of cases is estimated to reach 3.6 million (Varma et al., 2014).

Although many of the cases of amputations are trauma-related [2879 patients between 2011 and 2012 in the United States (Low et al., 2017)], the leading cause continues to be diabetes mellitus, with more than 1.5 million lower amputations worldwide, with 1.1 million being without prosthesis (Zhang et al., 2020).

### Peripheral Nerve Regeneration and Repair

**FIGURE 2:**
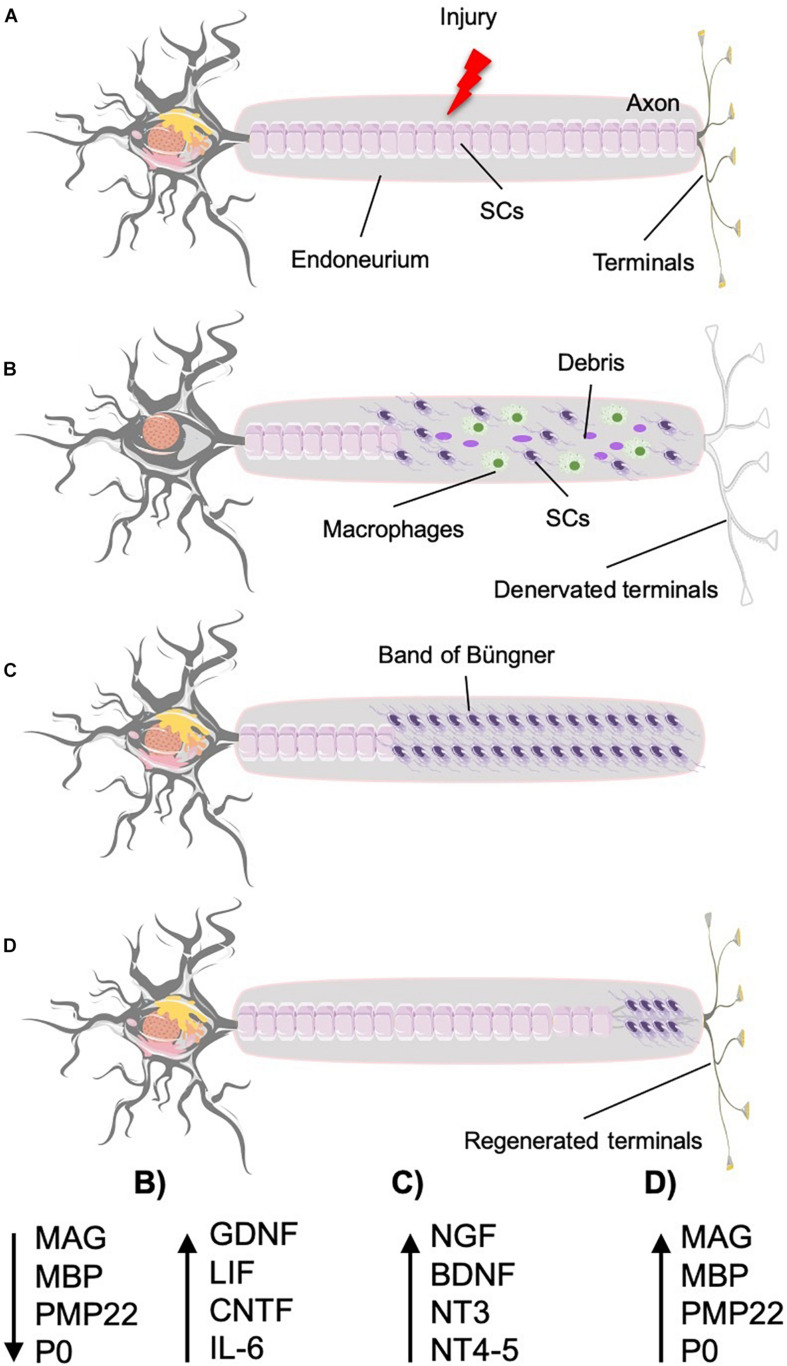
Nerve injury and regeneration under ideal conditions. Time course and biomolecular processes. **(A)** Normal neuron that suffers an injury (crush or section) that triggers the regeneration process. **(B)** The proximal part undergoes retrograde degeneration and chromatolysis. The distal part suffers Wallerian degeneration, provoking the denervation of the target tissue and leaving the endoneurium clean for the axon to regenerate (MAG, MBP, PMP22, and P0 levels decrease, whereas GDNF, LIF, CNTF, and IL6 production increases). **(C)** Schwann cells form bands of Büngner to guide the regenerated axons into the distal endoneural tube (with high levels of NGF, BDNF, NT3, and NT4–5). **(D)** The axon grows in contact with the surfaces of the Schwann cells toward the target organ. As the axons grow, the Schwann cells start the remyelination process, producing the myelin-associated molecules (MAG, MBP, PMP22, and P0 return to their initial levels).

In PNI, spontaneous repair occurs if the nerve, although damaged, has not been sectioned, with the outer membrane (epineurium) remaining intact regardless of the severity of the injury suffered by the internal structures, which can be damaged or even sectioned (Seddon, 1942; Sunderland, 1951; Menorca et al., 2013). Inside the nerve, the damaged Schwann cells change their phenotype, from myelinating to repairing one, and start secreting neurotropic and neurotrophic biomolecules, creating a facilitator environment that promotes the regeneration of the injured axons and guides them to their targets, sensitive or motor ones (Dubový, 2004; Eberhardt et al., 2006; Höke et al., 2006). Sensory/motor axon injury divides the neuron in two parts: the “proximal” (to the spinal cord), which comprises the soma, the part of the axon attached to the soma and the arborizations, and the “distal” (from the spinal cord), which comprises the segment of the axon detached from the soma together with their sensory/motor endings (Figure 2). The distal part of the nerve undergoes degeneration, whereas the proximal part starts growing and invading the space of the distal one, now left free. The growing fibers in the proximal part are attracted by the distal part and conveyed separately to their respective targets by the neurotropic and neurotrophic biomolecules secreted by the Schwann cells. These processes occur into the intact epineurium, which preserves the facilitator environment by retaining the secreted biomolecules close to the injured axons, and at the same time, it works as a conduit for the regenerating fibers, directing them toward the territory they have to reinnervate (see *The Need of an Ordered and Differentiated Regeneration of Sensory and Motor Fibers in Amputee People* for details).

**FIGURE 3:**
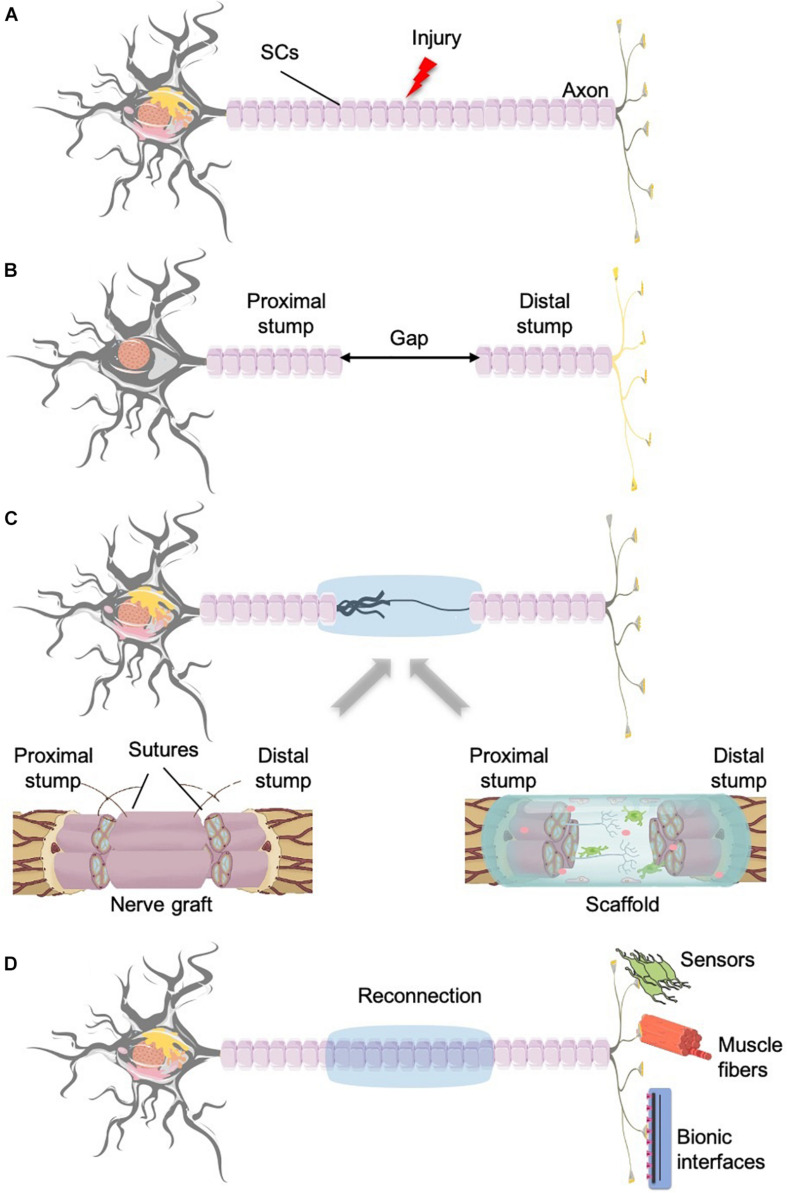
Nerve injury and regeneration with the aid of grafts or biomaterials scaffolds. **(A)** Neuron divided into proximal stump and distal part that innervates the target tissue. **(B)** After the damage takes place, the gap created between the two parts provokes that the regeneration cannot take place properly because of the lack of communication between the two stumps. **(C)** Different approaches to obtain the connection of the two stumps and the guidance of the growing axons: left, nerve graft; right, biomaterials and tissue engineering. **(D)** Once the reconnection of the two ends of the injured nerve is achieved, these neurons will be able to reinnervate the former target tissue, divided into sensors and muscles; or in the case that the target tissue has been lost, the reconnection with bionic interfaces.

Unfortunately, in the majority of the cases, PNI also implies nerve tissue losses, either due to the accident itself, or because of tissue removal during the surgical intervention. In these circumstances, to allow the neuroreparative processes, we must (artificially) restore the continuity of the epineurium and create a bridge for the intercommunication of the two ends of the sectioned nerve (Brattain, 2014; Grinsell and Keating, 2014; Faroni et al., 2015; Figure 3). State-of-the-art devices are tubular scaffolds (Pabari et al., 2014; Dalamagkas et al., 2016), grafts (Mackinnon et al., 2001; Brushart, 2011; Brattain, 2014; Dalamagkas et al., 2016) or tissue engineering bio-hybrids (Gonzalez-Perez et al., 2018).

### The Need of an Ordered and Differentiated Regeneration of Sensory and Motor Fibers in Amputee People

**FIGURE 4:**
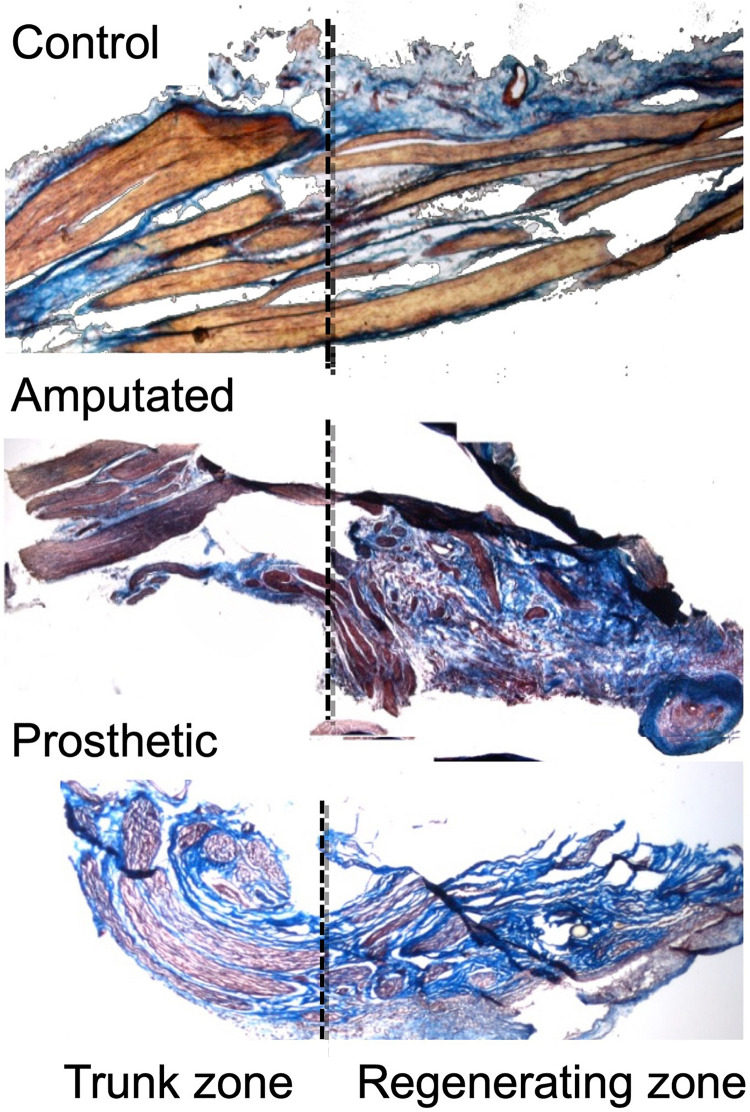
Effects of electric neurostimulation on the macroscopic anatomical structure of an amputated rat trigeminal peripheral nerve (Herrera-Rincon et al., 2011). Masson trichrome staining of nerve tissue from Control, Amputated, and Prosthetic animals. Nerve fibers can be detected as red-stained “bundles” crossing from left to right, surrounded by a blue-stained matrix (mostly collagen). In control nerve **(top)** the axons are organized in regular fascicles separated by connective tissue, whereas in the amputated nerve **(middle)**, the regular organization of the tissue is altered. However, whereas in amputated nerves, organization is completely lost with haphazardly arranged axons dispersed inside the connective tissue, in prosthetic nerve **(bottom)**, the fascicular organization is preserved. The importance of stimulation as a therapy to improve the regeneration of the growing fibers resides in the ability to achieve a guided and organized growth. Through Masson trichrome, we can observe the fascicular organization of the fibers, stained in brown and marked with arrow. In the left side of the images, the “trunk” is shown, the undamaged “proximal” area, which is in charge to activate the biomolecular regenerative processes. In the right, we can observe the regenerated fibers. Here we can see how when no treatment is performed, the tissue is disorganized, with a predominance of connective tissue (stained in blue, marked with arrow), whereas the treatment with electrical stimulation allows a more organized growth, maintaining the fascicular structure throughout the section (marked with arrow). Original magnification 100×. ©[2011] IEEE. Reprinted, with permission, from IEEE Proceedings.

Evidently, because of the lack of the distal end, repair is not possible in case of amputation. However, in amputees there are two very powerful reasons to achieve nerve regeneration patterns similar to those of non-amputated people, despite the lack of their (amputated) limbs. The first reason is to reduce the appearance of traumatic neuropathies and phantom limb pain, both of them caused by the aberrant regeneration of the peripheral nerves and by the formation of neuromas in the proximal stump. Indeed, aberrant regeneration and neuromas in sensory nerves could be reduced using selective electrical stimulation of the regenerating fibers (Herrera-Rincon et al., 2011; Figure 4). However, because almost all peripheral nerves are mixed, stimulation-based therapeutic approaches require the separated regeneration of sensory and motor axons. The second reason is to connect the sensory and motor nerves of the amputees to the sensors and actuators of the bionic neuroprostheses that are being available in the market, which demand well-differentiated and ordered sensory and motor axons to be separately guided and connected to the artificial sensors and actuators (Raspopovic et al., 2014; Micera, 2016; Lee et al., 2018; Günter et al., 2019; Deshmukh et al., 2020).

To achieve the differentiated regeneration also in amputee persons, the most intuitive and straightforward solution would be to reproduce in the amputated nerves the processes that naturally occur in damaged nerves with preserved epineurium and target organs, which means to create in the amputated member an artificial anatomical and biomolecular environment (a bio-hybrid medium) similar to the environment that favors the natural regeneration of the peripheral nerves. Unfortunately, and despite the enormous efforts dedicated in the last 70 years to this enterprise, differential regeneration of the sensory and motor fibers has not been yet totally achieved (Johnson et al., 2015; Anand et al., 2017; del Valle et al., 2018). Among the main reasons of this failure are the high complexity of the involved biomolecular processes and the variability of the employed experimental protocols, which make experimental data difficult to interpret. However, even more important is the total absence of a scientific article describing the way these biomolecular processes should be implemented by tissue and biomedical engineers to achieve the desired regeneration of the amputated nerves. In contrast, there is an extremely high number of scientific articles analyzing the regenerative processes.

Some of the commercially available upper-limb prostheses are Michelangelo (©Otto Bock, Germany), I-Limb Ultra (©Touch Bionics, United Kingdom) Bebionic (©RLS Steeper, United Kingdom), The Taska Hand (©Taska prosthetics, New Zealand), implantable at hand level or The LUKE arm, (©Mobius Bionics, LLC, United States), the DynamicArm elbow (©Otto Bock, Germany), and the Hero Arm (©Open Bionics, United Kingdom) implanted at shoulder level and allowing mobility over the patient’s head, being the last one the first three-dimensionally (3D) printed bionic arm. Commercially available lower limb prostheses include the C-Leg (©Otto Bock, Germany), the emPOWER ankle (©BionX Medical Technologies, Inc., United States), the Proprio Foot (©Ossur, Islandia), the BiOM ankle (©BiOM, United States), and the Elan Foot (©Endolite). All them are controlled using myoelectric signals coming from residual muscles in the amputee stump.

Much of research work is focusing on obtaining prosthetic sensory signals (Graczyk et al., 2018; Cuberovic et al., 2019), necessary not only to elicit the sensory perceptions of the natural limbs (George et al., 2019; Navaraj et al., 2019), but also to increase the control and the coordination of the prosthetic device without the need of visual and/or auditory signals, to identify the type of element being manipulated and to create proprioception (Micera et al., 2011; Zecca et al., 2017; Clemente et al., 2019; D’Anna et al., 2019; Sensinger and Dosen, 2020).

Patients with either intrafascicular and intraneural electrode implants show good proprioception and object identification capabilities (Micera et al., 2008; Di Pino et al., 2012). However, disruptive neuroprosthetic solutions require individual or quasi-individual nerve–electrode connections.

With the present article, we aim at offering a comprehensive compendium describing how to create neural regeneration devices, either scaffolds or tissue-engineered bio-hybrids, and how to implement the biomolecular processes that will foster the regeneration of the amputated peripheral nerves in a well-organized and guided manner. For this endeavor, we need a deep understanding of the biomolecular processes that underlie spontaneous nerve regeneration and of the spatiotemporal patterns of the expression of these biomolecules, as well as of their dynamic interactions over time, and to be able to implement them in a bio-hybrid biomaterial environment (Schmidt and Leach, 2003; Matsumoto and Mooney, 2006; Mikos et al., 2006; Biondi et al., 2008; Wang et al., 2009; Atala et al., 2012; Gu et al., 2014; Gonzalez-Perez et al., 2018).

In the following section, we will briefly review the biomolecular processes after PNI, and then we will go in depth to the specific processes to differentiate regeneration of sensory and motor fibers in amputee people.

## Spontaneous Regeneration and Repair After PNI

As mentioned previously, cellular and biomolecular processes in the proximal and the distal parts of the nerve are different. In both places, processes occur in two phases, a preparatory and a repair one. Any biomimetic device aiming at an ordered regeneration of the sensory and motor nerves has to implement both the anatomical structure of the distal part and the biomolecular processes that take place there.

### Preparatory Phase

In the proximal part, both the damaged axon and the myelin sheath that surrounds it undergo retrograde degeneration, approximately up to the first node of Ranvier (Figure 2; Fu and Gordon, 1997).

In the distal part, 48 to 96 h after the lesion starts the “Wallerian degeneration,” axon and myelin decompose, whereas both Schwann cells and macrophages recruited by these cells get rid of the debris and leave empty the whole distal endoneural tube (Stoll et al., 2002; Vidal et al., 2013; Chen et al., 2015; Barton et al., 2017), during the first 7 days after the injury (Caillaud et al., 2019). The empty endoneural tubes connect the injury region to the target organs, either sensors or muscle fibers (Eva and Fawcett, 2014; Romano et al., 2015). At this stage, we observe an increase of the production of proinflammatory molecules, such as cytokines [glial cell line–derived neurotrophic factor (GDNF), leukemia inhibitory factor (LIF), ciliary neurotrophic factor receptor (CNTFR), and interleukin 6 (IL6)], accompanied by a decrease in the production of myelin-associated molecules [myelin-associated glycoprotein (MAG), myelin basic protein (MBP), peripheral myelin protein 22 (PMP22), and myelin protein 0 (P0)] (Figure 2).

The physical structure of a biomimetic regenerative device should implement the above architecture of a high number of artificial conduits and be able to support the analogous neuroregenerative biomolecular processes.

### Repair Phase

**FIGURE 5:**
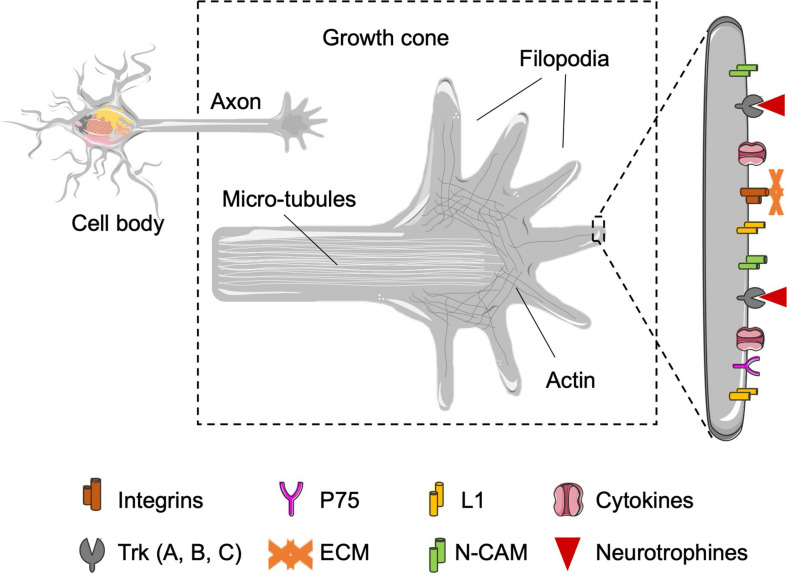
Representation of the growth cone in charge to interact with the Schwann cells and the ECM that surrounds the growing axons. Zoom of growth cone: representation of the growth cone in detail. The organization of the filopodia and the mobile structure in charge of interaction with the physical and chemical environment are supported by actin filaments accumulated in the proximal stump. Zoom of the filopodia: representation of the different molecules expressed in the growth cone. The molecules can be divided in three groups: (i) expression of neurotrophic factors receptors, for neurotrophins (Trk A, B, C, and p75) and neuropoietic cytokines [leukemia inhibitory factor receptor (LIFR), CNTFR…]; (ii) cell–cell junction molecules (N-CAM, L1), which will allow the interaction between the regenerating axons and the Schwann cells present in the bands of Büngner, in the distal zone, and (iii) molecules that bind to the ECM (integrins), which will act as a support for axonal guidance to its target tissue.

In the proximal part, 7 days after the damage, small branches sprout from the extreme of the sectioned axon and form the so-called “growth cone” (Figure 5; Ramón y Cajal, 1959; Bradke et al., 2012). It is a mobile structure with specialized receptors that recognize surfaces and molecules (Ertürk et al., 2007; Koch et al., 2012) and decides to which direction should grow the regenerating axon (Figure 5; Eva and Fawcett, 2014), guiding the regeneration.

In the distal part, the byproducts (molecules) of the degenerated axon and myelin stimulate the denervated Schwann cells to divide inside the endoneural tube; there, they get distributed along the longitudinal axis forming a column, the so-called bands of Büngner (Figure 2C; Pfister et al., 2011). The bands of Büngner create a bridge between the regenerating axons and the target tissue, also producing the necessary neurotrophic and neurotropic biomolecules to help axons to grow. In turn, Schwann cells in the tube attract the regenerating axon to enter the distal endoneural tube (Figure 2C; Lee and Wolfe, 2000). They do it by releasing neurotrophic and neurotropic molecules (Politis et al., 1982; Politis and Spencer, 1983) recognizable by the receptors present in the surface of the growth cone (Maggi et al., 2003; Gordon, 2009; Griffin et al., 2013; Faroni et al., 2015). It is worthy to underline that each endoneural tube attracts axons of its own (sensory or motor) modality (Ramón y Cajal, 1959).

Once introduced into the endoneural tube, the axon grows in contact with the surfaces of the Schwann cells toward the target organ. Upon reinnervation, Schwann cells return back to synthesize myelin and isolate the axon again (Grinsell and Keating, 2014). Axons that do not connect with their targets or do not reach the endoneural tube (as in the case of amputation) continue to grow but in a disorganized way and form an abnormal tissue structure, the neuroma (Grinsell and Keating, 2014).

A biomimetic regenerative device should incorporate a biomolecules-generating mechanism to release the same molecules with the same concentrations in space and time as the aforementioned Schwann cells. This is a highly dimensional problem, and it is important to mimic/duplicate the cues of endogenous regenerative microenvironment, when endogenous repair systems work.

### Differential Regeneration of Sensory and Motor Axons

The differential regeneration and guidance of sensory and motor axons toward their specific targets are determined by the molecules that are expressed by the Schwann cells of the distal endoneural tubes. Such molecules can be classified into two groups.

The first group includes five types of neurotrophic factors: axonal growth promoters (neurotrophins), neuropoietic cytokines, fibroblast growth factors, transforming growth factors, and insulin-like growth factors (Boyd and Gordon, 2003; Jin et al., 2009; Li et al., 2020).

The second group includes molecules integrated into the surrounding nerve tissue, mainly axonal growth promoters, such as molecules that facilitate and modulate cell–cell and cell–extracellular matrix (ECM) adhesion [glycoproteins such as neural cell adhesion molecule (N-CAM), N-cadherin, L1, integrins, etc.] (Seilheimer and Schachner, 1988; Smith et al., 1994; Franz et al., 2005; Saito et al., 2005, 2010; Gardiner et al., 2007; Tucker and Mearow, 2008; Gardiner, 2011; Anand et al., 2017) and molecules present in the ECM (fibrinogen, fibronectin, laminin, etc.) (Politis, 1989; Hammarberg et al., 2000; Zhang et al., 2003; Tucker and Mearow, 2008; Webber et al., 2008; Gardiner, 2011; Fudge and Mearow, 2013; Gonzalez-Perez et al., 2016; Table 1).

**TABLE 1:** Neurotrophic and neurotropic molecules that are expressed by the Schwann cells in the distal endoneural tubes.

Neurotrophic factors		
Axonal growth promoters	NGF, BDNF, NT3, NT4/5	Boyd and Gordon, 2003; Jin et al., 2009; Li et al., 2020
Transforming growth factors	GDNF	Allodi et al., 2012; Hoyng et al., 2014; Anand et al., 2017
Insulin-like growth factors	IGF1 e IGF2	Di Giulio et al., 2000; Brushart et al., 2013
Fibroblast growth factors	FGF2	Allodi et al., 2012; Brushart et al., 2013; Santos et al., 2016b
Neuropoietic cytokines	PTN, CNTF, IL6, LIF	Mi et al., 2007; Gordon, 2014
**Molecules integrated into the surrounding nerve tissue**		
Axonal growth promoters	N-CAM, N-cadherin, L1, integrins, etc.	Seilheimer and Schachner, 1988; Smith et al., 1994; Hammarberg et al., 2000; Franz et al., 2005; Saito et al., 2005, 2010; Gardiner et al., 2007; Tucker and Mearow, 2008; Gardiner, 2011; Anand et al., 2017
Extracellular matrix molecules	fibrinogen, fibronectin, laminin, etc.	Politis, 1989; Hammarberg et al., 2000; Zhang et al., 2003; Previtali et al., 2008; Tucker and Mearow, 2008; Webber et al., 2008; Gardiner, 2011; Fudge and Mearow, 2013; Zeng et al., 2014; Santos et al., 2016b

*Each endoneural tube contains a specific blend of such molecules depending on the type of its axon (i.e., sensory or motor). Moreover, blends change in time.*

With the only exception of nerve growth factor (NGF), there are no molecules favoring the growth of one type of neurons while inhibiting the growth of the other, which means favoring the growth of sensory fibers while inhibiting the growth of the motor ones, or *vice versa* (Wang et al., 1997; Boyd and Gordon, 2003; Pehar et al., 2004, 2006). Some of them strongly favor the regeneration of sensory fibers but have limited effect on the motor ones; others strongly favor the regeneration of the motor fibers, but they don’t display facilitatory effects on the sensory ones; finally, a third group of molecules favor the regeneration of both types of fibers, although the facilitating effect may be greater on one type of fibers than on the other (see Table 2 and references therein). Conversely, inhibition of their function through the administration of antibodies against them or against their receptors normally leads to inhibition of the regeneration process and also abnormal regeneration of the fibers (Table 2 and references inside). Several cocktails of these molecules have been designed and incorporated to scaffolds for peripheral nerve regeneration and repair with deceptive up-to-day results (Table 2; Johnson et al., 2015; Anand et al., 2017; del Valle et al., 2018). Doses of the most relevant neurotrophic factors, which have been tested to improve the molecular environment for the regenerating axons, are shown in Table 3.

**TABLE 2:** Effects of the supply (+) or depletion (−) of axonal growth-promoting molecules in the surrounding tissue of sensory (S) or motor (M) fibers.

Molecule	S+	M+	S−	M−
NGF	↑	↓	↓	↑
BDNF	↑	↑	↓	
NT3	↑	↑	↓	↓
NT4/5	↑	↑		
GDNF	↑	↑		
IGF1	↑	↑	↓	
IGF2		↑		
FGF2	↑	↑		
CNTF	↑	↑		
PTN	↑	↑		

*References: NGF: S+, (Apfel, 1999; Höke et al., 2006; Webber et al., 2008; Hoyng et al., 2014; Santos et al., 2016b; Anand et al., 2017); M+, (Wong et al., 1993; Wang et al., 1997; Boyd and Gordon, 2003; Pehar et al., 2004, 2006); S−, (Averill et al., 1995; Suzuki et al., 2016); M−, (Pehar et al., 2004, 2006). BDNF: S+, (Apfel, 1999; Fine et al., 2002; Höke et al., 2006; Allodi et al., 2012; Gordon, 2014; Grosheva et al., 2016); M+, (Funakoshi et al., 1993; Wong et al., 1993; Friedman et al., 1995; Kobayashi, 1996; Apfel, 1999; Al-Majed et al., 2000, 2004; Hammarberg et al., 2000; Terris et al., 2001; Boyd and Gordon, 2003; Allodi et al., 2012; Gordon, 2014; Hoyng et al., 2014; Santos et al., 2016b); S−, (Geremia et al., 2010). NT3: S+, (Apfel, 1999; Santos et al., 2016b; Anand et al., 2017); M+, (Funakoshi et al., 1993; Wong et al., 1993; Apfel, 1999; Hammarberg et al., 2000; Boyd and Gordon, 2003; Allodi et al., 2012; Anand et al., 2017); S−, (Hou et al., 2012; Wang et al., 2015); M−, (Wang et al., 2015). NT4/5: S+, (Apfel, 1999; English et al., 2005, 2011); M+, (Funakoshi et al., 1993; Wong et al., 1993; Friedman et al., 1995; Apfel, 1999; Boyd and Gordon, 2003; English et al., 2005, 2011). GDNF: S+, (Apfel, 1999; Keast et al., 2010; Allodi et al., 2012; Santos et al., 2016b; Anand et al., 2017); M+, (Apfel, 1999; Hammarberg et al., 2000; Boyd and Gordon, 2003; Allodi et al., 2012; Hoyng et al., 2014; Santos et al., 2016b; Anand et al., 2017; Ruven et al., 2018). IGF1: S+, (Kanje et al., 1989; Brushart et al., 2013); M+, (Li et al., 1994; Di Giulio et al., 2000); S−, (Kanje et al., 1989). IGF2: M+, (Brushart et al., 2013). FGF2: S+, (Grothe et al., 2006; Allodi et al., 2012; Brushart et al., 2013; Santos et al., 2016b); M+, (Allodi et al., 2012; Brushart et al., 2013; Santos et al., 2016b). PTN: S+, (Anand et al., 2017); M+, (Mi et al., 2007; Gordon, 2014). CNTF: S+, (Apfel, 1999); M+, (Wong et al., 1993; Apfel, 1999; Hammarberg et al., 2000). Up-oriented arrows (↑) indicate facilitation of the regeneration of these fibers, whereas down-oriented arrows (↓) indicate inhibition. No arrow indicates that this experimental condition has not been tested. Supply of any of the neurotrophic factors to the injured fibers, either sensory or motor, favors the regeneration of both fibers. NGF is the only exception.*

**TABLE 3:** Baseline protein levels of the most important molecules involved in the regeneration process and doses employed to obtain a favorable regenerative environment.

Nt Factor	Baseline protein levels	Experimental doses		
		Mixed nerves	Sensory nerves	Motor nerves
**Preclinical trials**				
NGF	211 pg/mg [Grosheva et al., 2016] 130 pg/mL [Shakhbazau et al., 2012]	0.8–1 μg/mL [Kemp et al., 2011; Santos et al., 2016a] 0.5 μg/mL [Chang et al., 2017; Li et al., 2018]	1 μg/mL [Kemp et al., 2011; Santos et al., 2016a] 20 μg/mL [Anand et al., 2017]	
BDNF	5 pg/mg [Grosheva et al., 2016] 14.8 pg/mL [Omura et al., 2005; Shakhbazau et al., 2012; Shakhbazau et al., 2012]	10 μg/mL [Kemp et al., 2011; Santos et al., 2016a] 10–100 μg/mL [Chang et al., 2017]		2 μg/mL [Kemp et al., 2011; Santos et al., 2016a] 20 μg/mL [Anand et al., 2017]
NT3	6 pg/mL [Shakhbazau et al., 2012] 100 pg/mg [Omura et al., 2005; Shakhbazau et al., 2012]	2 μg/mL [Kemp et al., 2011; Santos et al., 2016a]	1 μg/mL [Glazner et al., 1993]	0.5 μg/mL [Sterne et al., 1997]
GDNF	150 pg/mL [Shakhbazau et al., 2012]	20 μg/mL [Alsmadi et al., 2018]	20 μg/mL [Anand et al., 2017]	20 μg/mL [Anand et al., 2017]
IGF1	793 pg/mg [Grosheva et al., 2016]	50–100 μg/mL [Sullivan et al., 2008]	100 μg/mL [Apel et al., 2010]	100 μg/mL [Apel et al., 2010]
IGF2	39 pg/mg [Grosheva et al., 2016]	1 μg/mL [Caplan et al., 1999]	1 μg/mL [Glazner et al., 1993]	0.05 μg/mL [Near et al., 1992]
FGF	45 pg/mg [Grosheva et al., 2016]	0.025–0.5 μg/mL [Kemp et al., 2011; Santos et al., 2016a] 0.5 μg/mL [Li et al., 2018]	10 μg/mL [Lee et al., 2017]	100 μg/mL [Guzen et al., 2016]
CNTF	100 ng/mg [Grosheva et al., 2016]	300 μg/mL [Newman et al., 1996]	50 μg/mL [Dubový et al., 2011]	
PTN	20.2–24 pg/mL [Höke et al., 2006]	20 μg/mL [Alsmadi et al., 2018]	20 μg/mL [Anand et al., 2017]	0.1 μg/mL [Mi et al., 2007]
**Clinical trials**				
NGF	0.1 μg/kg [Apfel, 2002; Apfel, 2002; Li et al., 2020]			
FGF	2 ml [Apfel, 2002; Wu et al., 2011; Li et al., 2020]			

*The reference articles for this value are in square brackets.*

Although artificially delivered axonal growth-promoting molecules improve, in general terms, nerve regeneration and repair, they fail when they are used to differentiate the growth of sensory and motor fibers. This is due to the massive delivery from up-to-day devices (pumps, injections, releases from embedded biomaterials or biofunctionalized scaffolds, etc.), which neither simulate neither mimic the profile of the production of the neurotrophic factors during the regeneration. Even more important than the correct administration of the facilitator molecules is the timing of the delivery of such molecules. Timing determines if there will be an effect and which type of effect it will be. A very illustrative example is the administration of brain-derived neurotrophic factor (BDNF) and GDNF, two neurotrophic factors that, if delivered immediately after the injury, do not favor the regeneration of the nerve, while when administered after a brief period of adverse conditions for the regeneration process, their administration will have a clear favorable effect (Gordon, 2009). In PNI, what is important is timing for delivery of one or another molecule, but there is no “sensitive period” for inducing nerve regeneration after injury, typical in central nervous system injuries such as stroke (Dromerick et al., 2015; Zeiler et al., 2016; Ballester et al., 2019).

Summarizing, unsuccessful artificially guided differential regeneration of sensory and motor fibers is due to at least three main causes: (1) the delivery of neurotrophic factors acts on the whole nerve instead of on specific nerve fibers; (2) delivered doses are too high in comparison with the concentrations of the neurotrophic molecules observed in the nerve during the regeneration process; and (3) administration of the different biomolecules does not follow the time course of the concentrations of such molecules during the natural regeneration process.

## Design of Devices and Scaffolds for the Differentiation of Sensory and Motor Fibers

### Biological Principles

The above experimental data lead us to formulate the two biological principles that govern the regeneration of the peripheral nerves: (1) the separation of the fibers and their guidance toward their corresponding targets depend on the dynamics of the molecular gradients along the regeneration pathway (how the concentration of each molecule, at each point, varies with time), rather than on the concentration of the factors themselves; and (2) the molecules underlining these regenerative processes act at the microscopic level and not at the macroscopic one (they act at the level of individual fibers or fascicles of fibers, and not at the level of clusters of fascicles or of the whole nerve).

The design of novel approaches and devices for peripheral nerve regeneration and repair and, in particular, for the connection of amputee nerves to bionic sensory–motor prostheses should be driven by the above two principles.

### Current PNIs Approaches

In case of nerve sections with <5-mm-long gaps, the ideal PNI repair approach is the neurorrhaphy of the two stumps, suturing the individual fascicles one-by-one. In >5-mm-long gaps, the treatment consists of (i) the use of autografts and allografts or (ii) the implant of tubular biomaterials that reconnect the two stumps.

The gold standard are autografts, although they have several associated side effects, such as secondary surgery, donor site morbidity, size mismatch, and limited tissue availability. Commercial allografts, such as Avance^®^ Nerve Graft (Axogen, Inc., FL, United States), which consists of the ECM of a human nerve, without cellular or non-cellular debris (Karabekmez et al., 2009), avoid such effects; however, it is associated with the misdirection of the growing neurons and the necessity of immunosuppressant treatment.

Biomaterials to support reconnection and guidance of the regenerating axons present a series of specific characteristics that not only facilitate axon guidance but also allow acceptance of the implant by the nervous tissue like biocompatibility, biodegradability, mimetics of the host tissue, etc. Almost the totality of commercial devices for <30-mm-long PNIs consists of a hollow tubular scaffold that allows physical regeneration of the sectioned nerve through it. These scaffolds can be made by different biomaterials, such as porcine submucosa ECM, Axoguard^®^ Nerve Connector (Axogen, Inc., FL, United States); collagen I, NeuraGen^®^ (Integra LifeSciences Corporation, United States); polyglycolic acid, Neurotube^®^ (Synovis Micro Companies Alliance, Inc., AL, United States); and poly-DL-lactide-co-caprolactone and polyvinyl alcohol, Neurolac^®^ (Polyganics, Netherlands) (Tian et al., 2015; Costa Serrão de Araújo et al., 2017). Several improvements have been tested in animal models, including (i) fillings with hydrogels that favor axons regeneration; (ii) inclusion of topographic cues, like microfilaments/nanofilaments or groove patterns, to favor guidance and directionality by interacting with the growth cone; or (iii) incorporation of growth factors and supporting cells (Carvalho et al., 2019; Wang and Sakiyama-Elbert, 2019).

Guided axon regeneration in >30-mm-long gaps presents serious difficulties. In these cases, nerves lose their original topographic organization, which provoke the misdirection of the axons and the intermingled growth of the different types of neurons (Yi et al., 2019). Even though the guidance and regeneration of the neurons have been improved, axonal misdirection and innervation of the inappropriate target tissues are still unanswered clinical issues.

### Approaches for Differential Regeneration

Two main approaches have been used to guide a specific type of neuron to its originally innervating tissue: (1) use of biomaterials-built devices with two separated compartments (“Y-shaped” form scaffolds) to create separate molecular environments and thus achieve a separated regeneration of the sensory and motor neurons (Anand et al., 2017; del Valle et al., 2018) and (2) replacement of the classic single lumen tube by multichanneled scaffolds, each channel resembling the endoneurium (Tran et al., 2014) and creating independent molecular environments with different neurotrophic factors (Chang et al., 2017). 3D printing is boosting the development of novel biomaterial solutions and therapeutic approaches (Dinis et al., 2015; Dixon et al., 2018; Petcu et al., 2018). However, although the aforementioned developments envision a great future for PNI therapy and restoration of the sensory and motor functions to amputee people, the correct guidance and regeneration of the peripheral neurons to their target tissues are still an open problem.

To improve axon growth control, a more complex artificial molecular environment is necessary mimicking the biomolecular processes that guide natural axon regeneration. It is essential to achieve a complete control of the spatial and temporal dynamics of the release of the appropriate molecules, taking always into account the synergies between molecules.

### Design Principles for a Biomimetic Artificial Nerve

**FIGURE 6:**
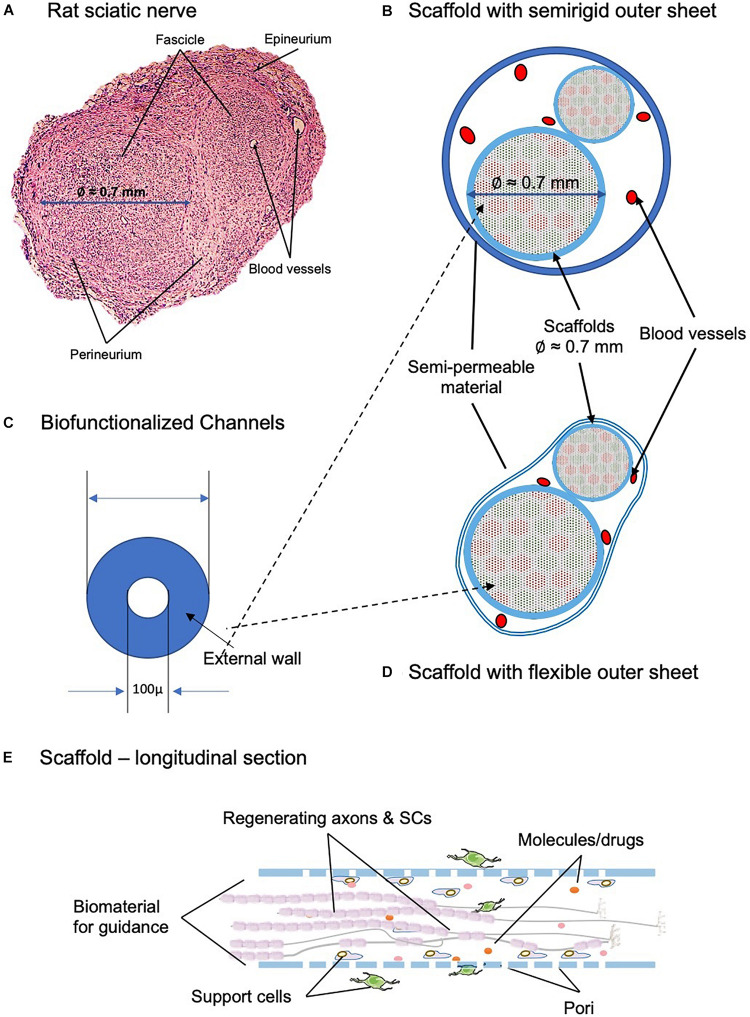
Desired biomimetic devices and scaffolds for neurotrophic biomolecules to be implemented in a future PNI-repair scaffold. **(A)** Transversal section of the rat sciatic nerve, with two fascicles and the different layers of connective tissue that organize the anatomy of the nerve. The differences on size between the fascicles can be observed. **(B,D)** Transversal section showing the 3D scaffold architecture, oriented to sensory–motor–guided regeneration; channels for sensory axons are in green, and motor in red. Scaffold of Ø 1.5 to 2.0 mm. The outer sheet can be both semirigid **(B)** or flexible **(D)**, depending on the area of the implant and the characteristics of the surrounding tissue that defines the most adequate option. **(C)** Magnification of the biofunctionalized channel. Channels of Ø 100 μm for the guidance of individual axons, sensory or motor ones. **(E)** Longitudinal section of the scaffold representing both the physical and chemical cues that determine the regeneration of the sectioned axons. The biomaterial provides the neurons with supporting cells, such as Schwann cells (SCs), molecules, and drugs. Furthermore, it acts as a substrate for the attachment of the growing axons.

Novel devices should be biomimetic in both the structure and the biomolecular environment they create around the regenerating axon. They should be built by hundreds or thousands of scaffolding structures, such as microtubes or high-performance fibers, each of which recreating the dynamic molecular gradients of the naturally regenerating nerves (Figure 6). However, it is worthy to note that nerve rewiring is not necessary to be perfect. Brain remodeling of the sensory and motor maps will correct deficiencies in wrong peripheral nerve connections and lead to a good level of sensory and motor functions of the patients (Merzenich and Jenkins, 1993; Panetsos et al., 2008).

At a microscopic scale, their internal channels, the artificial endoneural guides/tubes, will guide the growth of only a few axons, ideally only one (Figure 6). Each scaffold, preferably biofunctionalized with *ad hoc* biomolecules, should be capable of binding to the surface receptors expressed in the growth cone, as well as of releasing facilitator biomolecules with arbitrary time profiles.

Functional connection with bionic interfaces will allow communication and control of afferent and efferent signals between nervous tissue and artificial systems. Scaffolds should be produced according to the modality of their supported nerve, because, at the distal end, we need to place the establish contact between the motor axons and the actuators of the prosthesis. Correspondingly, the scaffolds guiding the sensory axons should allow establishing contact between the axons and the artificial sensors of the prosthesis.

### Implementation of Dynamic Gradients of Neurotrophic Factors for PNI Devices and Scaffolds

**FIGURE 7:**
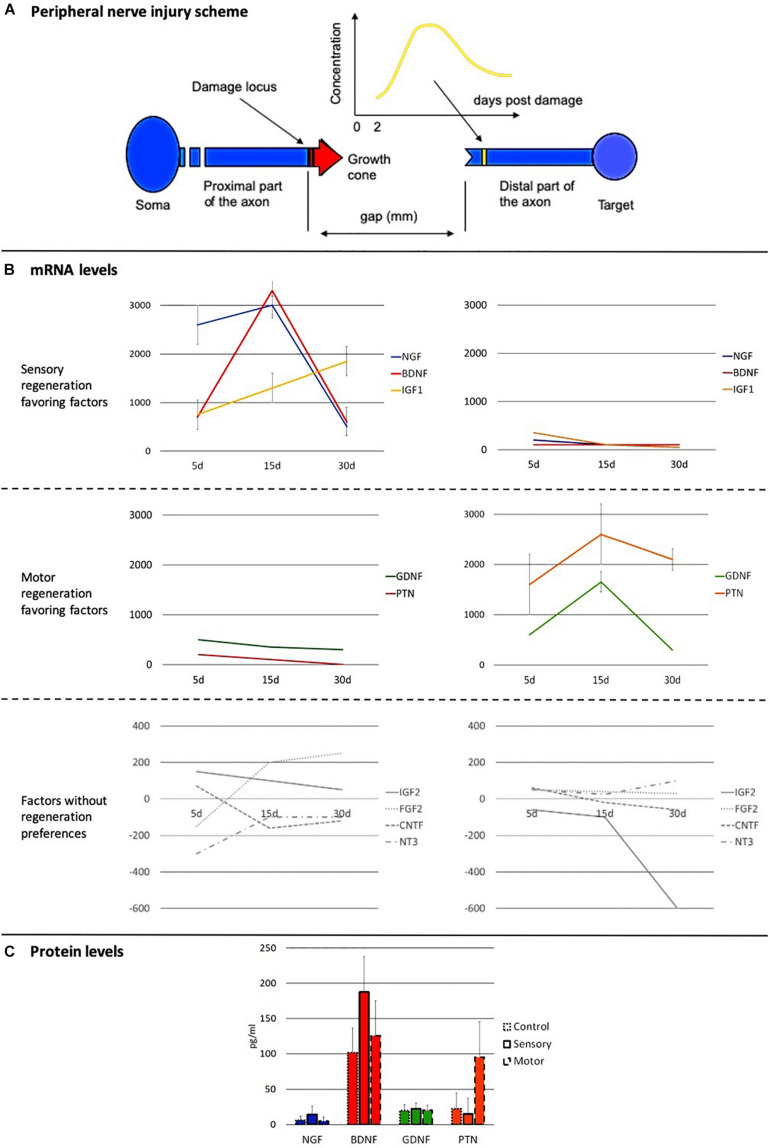
Dynamic behavior of the principal axon growth-promoting factors in the injured peripheral nerve. **(A)** Scheme of the peripheral nerve, of the damage region and of the growing process of the regenerating axons. A time-dependent expression profile of an axonal growth promoter molecule during the regeneration of the peripheral nerve at the site of the damage is also depicted. **(B)** mRNA expression profiles of the principal axonal growth promoter molecules in the distal stump, close to the injury locus. Left column: Expression profiles in injured sensory nerves. Right column: Expression profiles in injured motor nerves. Profiles are grouped according to their preferences in facilitating the regeneration of type of nerve: biomolecules mainly favoring the regeneration of sensory fibers (first row), biomolecules mainly favoring the regeneration of motor fibers (second row), and molecules not showing any preference for a specific type of fiber (third row). Data are represented in % change and in a logarithmic scale. **(C)** Protein levels (pg/mL) of the most important neurotrophic factors for the regeneration of both sensory and motor axons. Values are shown for uninjured nerves and for injured nerves 15 days after the injury [After (Höke et al., 2006)].

As stated before, in PNI without amputation, all the aforementioned biomolecules appear along the pathway of the growth cone, from the injury to the target organs. For each biomolecule, concentration values depend on the distance from the injury point and on the post-injury time (Figure 7A).

Molecules with strong expression during sensory axons regeneration are NGF, BDNF, and insulin-like growth factor 1 (IGF1); those with strong expression for motor fibers are pleiotrophin (PTN) and GDNF. Finally, neurotrophin 3 (NT3), ciliary neurotrophic factor (CNTF), IGF2, and fibroblast growth factor 2 (FGF2) are factors that do not show big differences in the regeneration of sensory and motor fibers.

Nerve growth factor, BDNF, and IGF1 are strongly involved in the regeneration of sensory nerves, showing very high concentrations since the beginning of the regeneration and very similar dynamics; in the motor nerve injuries, their expression is remarkably low (Figure 7B, first row). PTN and GDNF behave in a similar way but in favor of the motor axons (Figure 7B, second row). The two groups behave in similar manners, reaching their maximum concentrations 15 days after injury when promoting the regeneration, while the increase lasts only up to 5 days when the molecules are not favoring the growth of that type of fiber. All but NGF also exert a facilitating influence in the regeneration of nerves of the other sensibility, although in these cases the expression of these molecules is very limited on time and amount (Figure 7B). As for the molecules whose expression is not specific for the differential growth, such as NT3, CNTF, IGF2, and FGF2, they display neither intragroup nor intergroup similarities in their temporal behavior (Figure 7B, third row). It is important to highlight that the data represented in Figure 7 correspond to the mRNA and protein expression, which are very correlated, but not necessarily presenting the exact same profile.

Different methods have been studied for the administration of neurotrophic factors at the level of the sectioned nerve. Two main problems are the difficulty of a controlled administration for long periods of time and the diffusion of the neurotrophic factors far from the area of interest. The use of biomaterials allowed higher precision and greater control in the administration of the neurotrophic factors: osmotic pumps, hydrogels, polymeric microspheres, and the inclusion of the different molecules at the level of the tubular conduct implanted (Tajdaran et al., 2019). Factors are included within the lumen (hydrogels, nanofibers, and through the presence of cells that produce neurotrophic factors), in the conduit wall (included in the polymer or in microspheres), and at the surface (adsorbed or conjugated with other type of molecules) (Carvalho et al., 2019).

Biomaterials are important to be semipermeable, to allow the exchange of molecules between the growing axons and the environment. The size of the scaffold should be adaptable to the anatomy of each injured nerve. The presence of pores for a continuous exchange of molecules between the neural axes, the Schwann cells, and the surrounding tissue is important (Figure 6). Hydrogels seem to have the greatest future – thanks to their ability to release, the neurotrophic factors in a controlled manner, and to the presence and accumulation of high volumes of water – and to create physical conditions very similar to those of the nervous system (Li et al., 2018).

### Implementation of Neurotrophic Factors–Based Separation of Sensory and Motor Fibers

To guide fibers of the two modalities (sensory and motor) toward their dedicated cylinders, it is necessary, to first identify/select the sensory and the motor ones. A reasonable way to do that is to use several neurotrophins to favor the growing of one type of fibers in one direction, while preventing fibers of the opposite modality from growing with them, and *vice versa*. This is not a trivial question because there is only one inhibitory neurotrophic factor (NGF), and its action is exercised against the motor fibers (Table 2). This lack of selectively inhibiting biomolecules makes the separation of the fibers by biomolecular mutual repulsion–attraction complicated.

Conveying of sensory and motor fibers to these targets using only one inhibitory neurotrophin could be achieved by an intermittent short-term release of NGF close to the scaffolds dedicated to the sensitive fibers, combined with a timely release of PTN and GDNF from the interior of the motor channels. Discrete NGF amounts should diffuse in the scaffold and attract sensory fibers while blocking the motor ones; NGF deactivation should leave motor fibers free to start growing and get attracted by the neurotrophins-releasing motor channels. NGF activation/deactivation has to operate at the microscopic level (at the level of the channels of the scaffold, see section “Repair Phase”), creating a very short-term, high-amplitude attraction gradient (biomolecules concentrations). This NGF gradient would correlate with the NGF mRNA expression, which is the fastest molecule that reaches a high level of expression.

The importance of the neurotrophic factors’ administration goes further than just guiding the growth of the regenerating neurons. The function that this type of molecules carries out is well known, in the myelination process, during development (Piirsoo et al., 2010), and in the case of remyelination, once the axons reinnervate their target (Chan et al., 2001; Acosta et al., 2015; KhorshidAhmad et al., 2016; Razavi et al., 2017, 2018). This type of molecules is involved in the modification of the Schwann cells into a myelinating phenotype, once the regeneration is completed, activation that takes place via p75 receptor (Cosgaya et al., 2002; Notterpek, 2003; Xiao et al., 2013).

### Implementation of Substrate-Related and Other Facilitator Biomolecules

In the design of a biomimetic device, facilitator molecules integrated into the surrounding tissue, as well as attracting biomolecules produced by the distal end, should also been taken into account. Among the former, the most promising are fibronectin and laminin, and both can be used for the regeneration of sensory and motor fibers, although the preference of the fibronectin is slightly higher for motor, and the preference of laminin is for the sensory (Tucker and Mearow, 2008; Gonzalez-Perez et al., 2013, 2016). Factors integrated in the surrounding nerve tissue, which favor the binding of neurons to the substrate or cell–cell binding (mainly glycoproteins such as integrins, N-CAM, N-cadherins, or L1) (Seilheimer and Schachner, 1988; Smith et al., 1994; Hammarberg et al., 2000; Franz et al., 2005; Saito et al., 2005, 2010; Gardiner et al., 2007; Tucker and Mearow, 2008; Gardiner, 2011; Anand et al., 2017); ECM molecules (fibrinogen, fibronectin, or laminin) (Politis, 1989; Hammarberg et al., 2000; Zhang et al., 2003; Previtali et al., 2008; Tucker and Mearow, 2008; Webber et al., 2008; Gardiner, 2011; Fudge and Mearow, 2013; Zeng et al., 2014; Santos et al., 2016b); and molecules that act as chemorepellents or chemoattractants (semaphorins, ephrins, and netrins) (Tessier-Lavigne and Goodman, 1996; Dickson, 2002; Wang et al., 2013; Kaselis et al., 2014; Alto and Terman, 2017).

Last but not least, more molecules are produced at the distal end of the damaged nerve that serve to attract regenerating axons or to create an attractive substrate inside the distal endoneural tube (Figure 5).

In the case of biomaterial engineering and tissue engineering, these factors can serve the same functions that they perform in natural tissue, which is to facilitate the adhesion of axons and stimulate their growth, being their incorporation necessary when the artificial material is not attractive to axons.

## Discussion

A well-differentiated and ordered regeneration of the sensory and motor fibers is mandatory in all PNI cases: short/non-gap injuries, large gap injuries, amputations, neuropathies of traumatic etiology, phantom limb pain, etc. Clinical problems such as incomplete regeneration, impossibility of achieving differential growth of sensory and motor nerves, etc., are all due to the fact that current therapies (biomolecules, bio-hybrid scaffolds, etc.) all act at the level of the entire nerve, and its action cannot be transferred to individual axons or fiber fascicles (Du et al., 2018; Yi et al., 2019). Any biomolecule showing affinity for one type of fiber under spontaneous PNI repair, if applied at the microscopic level (individual axons level), loses its effectivity if it is applied at macroscopic level (entire peripheral nerve). Advanced devices and novel biomaterials at the microscopic level are required for the implementation of the desired functionalities based on neurotrophic factors and facilitator biomolecules.

From the previous discussion, the basic requirements for these novel biomaterials can be inferred at three levels: geometrical, mechanical, and biochemical. The size of the axons and the fascicles implies that the cross-sectional size of the scaffold should be in the range between a few and 100 μm, whereas the longitudinal size should be in the range of millimeters and even centimeters. This geometry, in turn, possesses a heavy constraint on the mechanical performance of the biomaterial, because it must sustain the surgical process, as well as the in-service life. In addition, the material must be compatible with the neurotrophic factors and adherent facilitator biomolecules.

Most present solutions cover some, but not all of these three requirements. Both natural and artificial materials are used for repairing PNIs (Aijie et al., 2018; Boni et al., 2018). Present commercial solutions are based on poly(glycolic acid) (Neurotube) and poly(D,L-lactide-co-e-caprolactone)–based (Neurolac) (Du et al., 2018), but other solutions were explored based on polylactic acid (Zeng et al., 2014), polylactic glycolic acid (Xue et al., 2012), and polyethylene glycol (Liu et al., 2015). Other proposals based on artificial materials employ electrical conductive polymers, such as PANi (Xu et al., 2016) and indium phosphide (InP) (Gautam et al., 2017), or carbon-based materials (Boni et al., 2018), such as graphene and carbon nanotubes. The use of artificial materials faces a number of problems. Thus, their biocompatibility tends not to be optimal, and most artificial materials cannot be functionalized. Natural materials tend to be more biocompatible, and consequently, these materials were also used as scaffolds for PNIs. Among the natural materials, solutions were proposed based on collagen (Mackinnon and Lee Dellon, 1990; Archibald et al., 1995) (NeuraGen, NeuroMatrix, and NeuraWrap), gelatin (Ghasemi-Mobarakeh et al., 2008; Zhu, 2010), hyaluronic acid (Song et al., 2014; Kondyurin et al., 2017), alginate (Sitoci-Ficici et al., 2018), chitosan (Wang et al., 2006), and keratin (Apel et al., 2008). However, most of these materials are difficult to be processed in scaffolds with the geometry and mechanical properties indicated above.

The nervous system has a superior level of complexity that demands sophisticated scaffolds and architectures, as well highly tolerable biomaterial formulations. Compatibility and toxicity still are a remaining concern that has not been completely resolved for an immense number of materials. For many tissues, classical biomaterials may result in feasible strategies that in general produce good positive outcomes (Green and Elisseeff, 2016). But neurobioengineering strategies are intricate because of the restrictive conditions of the nerve microenvironment. For example, certain byproducts derived from a classic material such as the hyaluronic acid are immunogenic and may trigger inflammation (Tesar et al., 2006). Deposits of hyaluronic acid may accumulate in demyelinated lesions preventing axon remyelination and functional rewiring (Back et al., 2005).

Among the different biomaterial formats, hydrogels-based formulations have been engineered and widely used to construct porous conduits to favor axonal guidance providing suitable environments or reconnect two peripheral nerve ends (Tao et al., 2017). Hydrogel conduits have also been assessed to advance in the resolution of other clinical problems of neurological origin, e.g., reconstruction of intraspinal neural circuits in spinal cords injuries (Marchini et al., 2019) or reconstruction of neurodegenerated nigrostriatal pathways in Parkinson disease (Struzyna et al., 2018). Considering that not all biomaterials accomplish for the strict requirements of the nervous system, silk appears as a very promising solution (Zhang et al., 2012). Because of its high biocompatibility and excellent mechanical properties (Heim et al., 2009), silk is currently being explored for the development of many new therapies (Vepari and Kaplan, 2007; Fernández-García et al., 2018). Furthermore, the possibility of producing regenerated silk fibers [artificially spun fibers, from a solution of natural silk protein (Madurga et al., 2017b)] led to the generation of high-performance silk fibroin fibers with a wide range of geometries, which, in addition, can be functionalized (Madurga et al., 2017a). The development of adequate materials for scaffolds that can be used for nerve repair, bridging gaps in lesioned nerves, will require a very intensive research program. However, the present range of available materials in combination with a deeper understanding of the biology of the natural repair processes opens new and promising perspectives for the development of these therapies.

## Conclusion

Current therapies are not effective and do not achieve a good regeneration because they act at the level of the entire nerve and not at the level of neuronal fiber or group of neuronal fibers and because they do not take into account the synergies between neurotrophic factors and their dynamic expressions over time and in the space. For the development of bio-hybrid materials for the correct regeneration of PNI, effective and reliable solutions must (i) work at microscopic level and (ii) take into account the molecular mechanisms of the regeneration and functional reinnervation of the sensory and motor nerves in both the proximal and the distal parts of the severed nerve.

## Data Availability Statement

The original contributions presented in the study are included in the article/supplementary material, further inquiries can be directed to the corresponding author.

## Author Contributions

AG-D and NJ-D: bibliographic research, information synthesis, writing the manuscript, and equal contribution. IO-Z: bibliographic research. GG, JP-R, DG-N: biomaterials aspects, critical DGN and manuscript revision. FP: manuscript design, information synthesis, supervision, and writing the manuscript. All authors contributed to the article and approved the submitted version.

## Conflict of Interest

The authors declare that the research was conducted in the absence of any commercial or financial relationships that could be construed as a potential conflict of interest.

## Abbreviations

BDNF: brain-derived neurotrophic factor
CNTF: ciliary neurotrophic factor
CNTFR: ciliary neurotrophic factor receptor
ECM: extracellular matrix
FGF2: fibroblast growth factor 2
GDNF: glial cell line–derived neurotrophic factor
IGF1: insulin-like growth factor 1
IGF2: insulin-like growth factor 2
IL6: interleukin 6
InP: indium phosphide
LIF: leukemia inhibitory factor
LIFR: leukemia inhibitory factor receptor
MAG: myelin-associated glycoprotein
MBP: myelin basic protein
N-CAM: neural cell adhesion molecule
NGF: nerve growth factor
NT3: neurotrophin 3
NT4/5: neurotrophin 4/5
P0: myelin protein 0
p75: low-affinity nerve growth factor receptor
PANi: polyaniline
PMP22: peripheral myelin protein 22
PNI: peripheral nerve injury
PNS: peripheral nervous system
PTN: pleiotrophin
Trk: tropomyosin receptor kinase.
